# Improvement of endocrine and metabolic conditions in patients with polycystic ovary syndrome through acupuncture and its combined therapies: a systematic review and meta-analysis

**DOI:** 10.1080/07853890.2025.2477295

**Published:** 2025-03-17

**Authors:** Tianyu Wu, Yiwei Liu, Fanjing Kong, Jinqun Hu, Yu Liu, Jie Yang, Jiao Chen

**Affiliations:** ^a^ School of Acupuncture-Moxibustion and Tuina, Chengdu University of Traditional Chinese Medicine, Chengdu, China; ^b^ Rehabilitation Medicine Center and Institute of Rehabilitation Medicine, West China Hospital, Sichuan University, Chengdu, Sichuan, PR China; ^c^ School of Health Preservation and Rehabilitation, Chengdu University of Traditional Chinese Medicine, Chengdu, China

**Keywords:** Polycystic ovary syndrome, PCOS, acupuncture, meta-analysis, data mining

## Abstract

**Background:**

Polycystic Ovary Syndrome (PCOS) is a common endocrine disorder among women of reproductive age that significantly impacts their reproductive health. Acupuncture and its combined therapies may have beneficial effects on the endocrine and metabolic states of women with PCOS. This systematic review and meta-analysis evaluated the treatment effects and potential mechanisms of acupuncture and its combined therapies compared to oral metformin in treating PCOS patients.

**Methods:**

The evaluation focused on three sets of outcomes: hormonal indicators, metabolic indicators, and body weight indicators. Studies that involved additional therapies beyond the specified interventions or included patients with other diseases were excluded. Additionally, data mining methods were used, including frequency statistics to analyze the frequency of acupuncture points and the meridians involved, and the Apriori algorithm to perform association rule analysis for the most effective interventions.

**Results:**

The study included 46 articles (51 studies) involving six interventions: acupuncture combined with metformin, acupuncture treatment, acupuncture with Chinese herbal medicine and metformin, acupuncture with Chinese herbal medicine, acupuncture combined with cupping, and auricular acupuncture combined with metformin showed significant improvements in all evaluated indicators. Data mining revealed the Stomach meridian of foot yangming was the most frequently used, and the most commonly used combination of points included CV4, SP6, and ST36.

**Conclusions:**

This study suggests that acupuncture and its combined therapies may benefit PCOS. However, risk of bias and heterogeneity observed were noted. Future high-quality, rigorously designed randomized controlled trials are needed to confirm these findings and provide stronger clinical recommendations for acupuncture in PCOS treatment.

## 1. Introduction

Polycystic ovary syndrome (PCOS) is a disease caused by metabolic dysfunction and endocrine system malfunction. It is predominantly characterized by irregular menstrual cycles or even amenorrhea, ovulation dysfunction, hyperandrogenism and polycystic ovaries[1,2]. PCOS is predominantly observed in women of reproductive age [3]. Globally, it is estimated that between 6% and 10% of women in this demographic are afflicted with PCOS, which is a prevalent cause of infertility in this population [4,5]. Clinically, patients with PCOS are frequently found to exhibit symptoms such as obesity and insulin resistance (IR). Research indicates that 80% of women with PCOS also suffer from IR, and 30% to 70% of women with PCOS are obese [6–8]. As the disease progresses, a significant proportion of these patients may develop type 2 diabetes and cardiovascular diseases [9–11]. Additionally, research has identified an increased risk of depression among women with PCOS, which significantly impacts their physical and mental health [12,13].

The pathogenesis of PCOS is complex and has not yet been fully defined. Hyperandrogenism caused by IR is considered a major potential pathological mechanism of PCOS [14]. In clinical practice, the treatment of PCOS predominantly involves medication, complemented by exercise and dietary interventions. Currently, commonly used medications include oral contraceptives, aromatase inhibitors, estrogen receptor modulators, and insulin sensitizers, among others. However, while these drugs can improve some symptoms of PCOS, they have not yet achieved the desired therapeutic effect. In addition, medication is often considered to be accompanied by significant side effects such as diarrhea and vomiting. Therefore, finding suitable treatments for PCOS is still a pressing issue. Acupuncture, a therapy from traditional Chinese medicine (TCM), regulates the internal physiological functions by stimulating specific parts of the body surface [15]. Due to its positive effects on disease treatment, acupuncture has increasingly attracted global attention from scholars and clinicians. Currently, acupuncture is widely used in the treatment of reproductive system-related diseases, including PCOS. An epidemiological report from the United States indicates that 22% of infertility patients have tried acupuncture as a treatment option [16]. Clinical trials focusing on acupuncture for reproductive health are actively being conducted in multiple countries and have already shown preliminary success [17]. Previous studies have indicated that acupuncture, when used as an adjunctive therapy, can effectively improve PCOS-related conditions, and its combination with metformin has been shown to significantly increase pregnancy rates in PCOS patients [18,19]. However, some scholars still believe that the experimental evidence is insufficient to support the efficacy of ­acupuncture in treating PCOS, and whether acupuncture has a therapeutic effect on PCOS remains ­controversial [17].

Currently, acupuncture and its combined therapies for the treatment of PCOS have been subjected to a large number of clinical trials and many scientific papers have been published [20–22]. Systematic analysis and summary of these studies are essential for drawing comprehensive and insightful conclusions regarding the efficacy of acupuncture and its combined therapies on PCOS. Numerous systematic reviews have explored the efficacy of acupuncture for polycystic ovary syndrome. However, these studies often lack strict standardization of control groups, which could lead to biased results and undermine the credibility of their conclusions [23]. Furthermore, some reviews include only a small number of studies and focus primarily on outcomes such as pregnancy and live birth rates, failing to provide a comprehensive systematic evaluation of the treatment’s effects [19]. Therefore, this study applied meta-analysis and data mining techniques to conduct a comprehensive investigation of acupuncture and its combined therapies in clinical trials on PCOS patients. We thoroughly assessed the effects of acupuncture and its combined therapies on hormone indicators, metabolic indicators, and weight indicators in PCOS patients, compared to those treated with metformin alone. Additionally, the mechanisms of treatment were extensively discussed. In addition, subgroup analyses were carried out to assess different interventions individually, with the aim of comparing their effects on improving the aforementioned indicators. Meanwhile, commonly used acupuncture points for treating PCOS were identified by analyzing the frequency, meridian categorization and association rule analysis of acupuncture points in the included articles. This study comprehensively summarizes and analyzes the therapeutic effects of acupuncture and its combined therapies on PCOS. These results not only provide data support and evidence of the therapeutic potential of acupuncture and its combined therapies in the treatment of PCOS, but also provide references for subsequent in-depth studies and clinical applications.

## 2. Methods

### 2.1. Review protocol

This study adheres to the Preferred Reporting Items for Systematic Reviews and Meta-Analyses (PRISMA) guidelines and the Cochrane Collaboration’s requirements, and it is registered with PROSPERO (CRD42024506359).

### 2.2. Search strategy

Using “Acupuncture” and “Polycystic Ovary Syndrome (PCOS)” as keywords, a search strategy combining subject terms and free terms was employed to search across seven databases: PubMed, Cochrane Library, Embase, Web of Science, China National Knowledge Infrastructure (CNKI), China Science and Technology Journal Database (VIP), and Wanfang Data Information Site (see Supplementary Table 1 for the search strategy). Articles were retrieved from the inception of each database until 13 September 2023. No restrictions on the language of the retrieved literature. If an article includes multiple, relatively independent controlled studies comparing experimental and control groups, each study that meets our inclusion criteria is counted individually in the total number of included studies. The selection process involved screening the titles, abstracts, and full texts of the retrieved articles using EndNote software. This screening was independently carried out by Tianyu Wu and Fanjing Kong. Any discrepancies encountered were discussed and resolved by Jinqun Hu and Yu Liu.

### 2.3. Inclusion and exclusion criteria

#### 2.3.1. Inclusion criteria

1. Population: individuals with a confirmed diagnosis of PCOS based on the Rotterdam criteria (ESHRE/ASRM 2004), or the Androgen Excess Society criteria for PCOS [2,24]. There are no restrictions on patient race or disease duration;
2. Intervention: the treatment group patients received either acupuncture alone or acupuncture combined with other therapies; the control group patients only received metformin;
3. Outcome: hormonal indicators, including luteinizing hormone (LH), luteinizing hormone/follicle-producing hormone (LH/FSH), and testosterone (T); and metabolic indicators, including fasting glucose (FPG), fasting insulin (FINS), and Homeostatic Model Assessment of Insulin Resistance (HOMA-IR). Articles were included if they contained at least one of these outcomes.

#### 2.3.2. Exclusion criteria

1. Non-randomized controlled trials;
2. Patients with other diseases such as non-classical 21-hydroxylase deficiency, hyperprolactinemia, Cushing’s disease, androgen-secreting tumors, diagnosed diabetes mellitus or pre-diabetes mellitus, renal or hepatic disease, etc.

### 2.4. Data extraction and quality assessment

The following information was extracted from the included articles: (1) Basic information (first author, publication year, sample size); (2) Patient information (average age, duration of illness); (3) Intervention measures (intervention method, intervention time, intervention frequency, treatment duration, acupoint selection); (4) Experimental results (hormonal indicators: LH, LH/FSH, T; metabolic indicators: FPG, FINS, HOMA-IR; weight indicators: BMI, WHR). When a study includes multiple results, consider them as independent data.

The risk of bias in the included studies was assessed using the Cochrane Risk of Bias tool for RCTs [25]. This assessment covered seven domains: Random sequence generation, Allocation concealment, Blinding of participants and personnel, Blinding of outcome assessment, Incomplete outcome data, Selective reporting and Other bias. If a report met the criteria in any of these domains, it was considered low risk; if it did not meet the criteria, it was considered high risk; if the details in the report were insufficient to properly assess the risk of bias, it was judged as “unclear”. The risk of bias was evaluated independently by two authors, and any disagreements were resolved through discussion with a third researcher.

The quality of evidence for each outcome was assessed by two independent researchers (Tianyu Wu and Fanjing Kong) using the evaluation (GRADE) system. The quality of evidence was rated as “high”, “moderate”, “low” or “very low” based on the recommended rating criteria in GRADE. Two researchers cross-checked the results after assessing the quality of evidence. Any disputes were resolved through discussion or consultation with a third researcher. The data extraction and quality assessment were independently carried out by Tianyu Wu and Fanjing Kong. Any discrepancies encountered were discussed and resolved by Jinqun Hu and Yu Liu.

### 2.5. Publication bias

Stata 18 was utilized to conduct Egger’s test, and funnel plots were generated. If any indicators are found to exhibit high publication bias, a sensitivity analysis will be performed to identify the source of bias.

### 2.6. Statistical analysis

The extracted data are continuous variables, represented by mean and standard deviation (SD), and analyzed by calculating the mean difference (MD) or standard mean difference (SMD). The heterogeneity of each study’s results is determined through the Chi-squared test and the I^2^ statistic, where *I^2^* ∈ (0, 40%) indicates that heterogeneity might be insignificant; *I^2^* ∈ (30%, 60%) indicates moderate heterogeneity; *I^2^* ∈ (75%, 100%) indicates high heterogeneity. Based on the results of heterogeneity, a random-effects model or a fixed-effects model is chosen, with the random-effects model being selected in cases of high heterogeneity. *p* < 0.05 is considered statistically significant. Analyses are conducted using Review Manager 5.3 software.

### 2.7. Data mining

To conduct an in-depth analysis of acupuncture points, this study uses the Python programming language (software: Spyder 5.4.1) and libraries including pandas for data processing and matplotlib.pyplot for visualization. Frequency and meridian analysis were conducted using statistical methods to determine the most commonly used acupuncture points and meridians. In terms of association rule analysis, this study employs the Apriori algorithm to generate frequent itemsets and association rules, with the minimum support set at 0.05 and the minimum confidence set at 0.1. By setting the number of antecedents and consequents, the study explores combinations of acupuncture points with strong associations.

## 3. Results

### 3.1. Study selection

**Figure 1.**
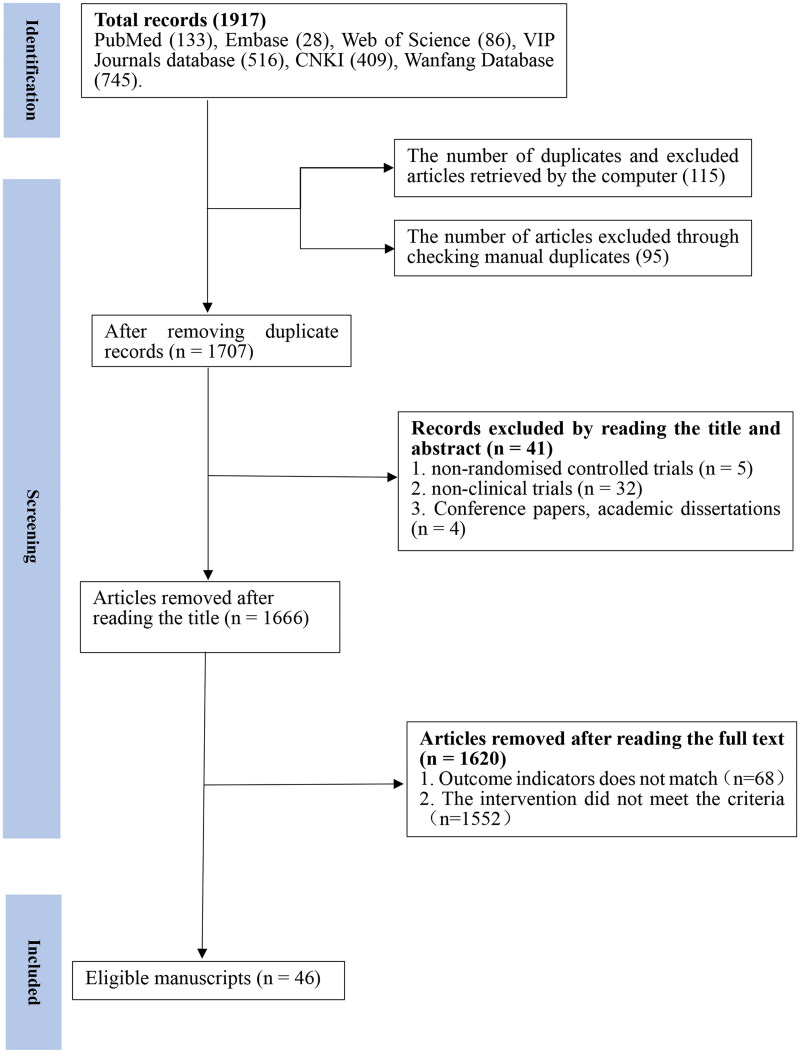
Flow diagram of the study selection process.

A total of 1917 articles were retrieved from seven databases, and after removing duplicates (*n* = 210), 1707 articles were finalized. After reviewing the titles and abstracts, 1693 papers were excluded for at least one of the following reasons: (1) The intervention did not meet the criteria (*n* = 1552); (2) non-RCT articles (*n* = 5); (3) non-clinical trials (*n* = 32); (4) Conference papers, academic dissertations (*n* = 4). Finally, after reading the full texts, 46 articles (encompassing 51 studies) were included for analysis (Figure 1).

#### 3.1.1. Study characteristics

1. Participants
  A total of 4181 participants were included, with ages ranging from 17 to 44 years. All participants met the Rotterdam criteria (ESHRE/ASRM 2004), or the Androgen Excess Society criteria for PCOS.
2. Intervention
  Among the 51 studies (46 articles), 16 studies (31.4%) reported acupuncture treatment [26–41], 21 studies (41.1%) reported acupuncture combined with metformin [31,33,34,39,42–58], nine studies (17.6%) reported acupuncture with Chinese herbal medicine [59–67], three studies (5.9%) reported acupuncture with Chinese herbal medicine and metformin [62,68,69], one study (2.0%) reported acupuncture combined with cupping therapy [70], and one study (2.0%) reported auricular acupuncture combined with metformin [71]. Most studies employed a treatment duration of three months; nine studies reported six months, one study reported four months, and one study did not specify the treatment duration. The frequency of acupuncture treatment varied substantially, ranging from once every 15 days to once per day.
3. Comparator
  All control groups received metformin alone.
4. Outcomes

The reported outcomes were heterogeneous. Among the 51 included studies, 36 reported metabolic indicators: 29 reported FPG, 27 reported FINS, and 34 reported the HOMA-IR. In terms of hormonal parameters, 32 trials offered relevant information: 34 reported LH, 29 reported the LH/FSH, and 37 reported the T. In total, 35 studies reported body weight indicators: 39 reported BMI and 21 reported WHR. The characteristics of these studies are detailed in Supplementary Table 2.

#### 3.1.2. Quality assessment of literature

**Figure 2.**
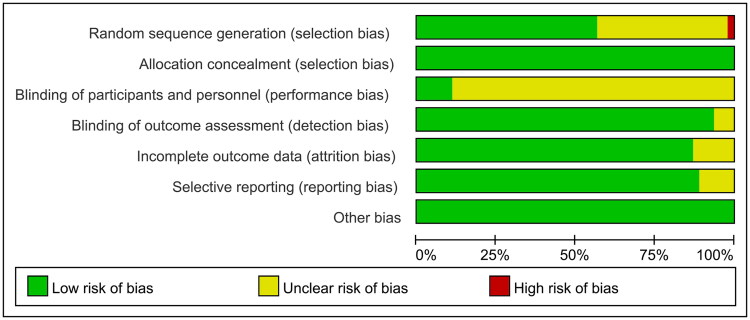
Assessment of literature quality using the Cochrane risk of bias tool for RCTs.

A total of 46 articles were reviewed (Supplementary Figure 1). Of these, 28 articles (60.9%) reported random sequence generation, all 46 articles (100%) reported allocation concealment, five articles (10.9%) reported blinding of participants and personnel, 44 articles (95.7%) reported adequate blinding of outcome assessment, 41 articles (89.1%) reported adequately addressing incomplete outcome data, and 42 articles (91.3%) reported adequately addressing selective reporting (Figure 2).

According to GRADE (Supplementary Table 3), the quality of evidence ranged from low to moderate. The inconsistency was the main reasons for downgrading. FINS and WHR were classified as low quality, as their *I^2^* values exceeded 90%.

### 3.2. Hormonal indicators

#### 3.2.1. LH

A total of 34 studies included in this study assessed the LH indicator. Among them, 16 studies involved acupuncture combined with metformin, 10 studies focused on acupuncture alone, six study investigated acupuncture with Chinese herbal medicine, one study involved acupuncture with Chinese herbal medicine and metformin, and one study investigated acupuncture combined with cupping.

**Figure 3.**
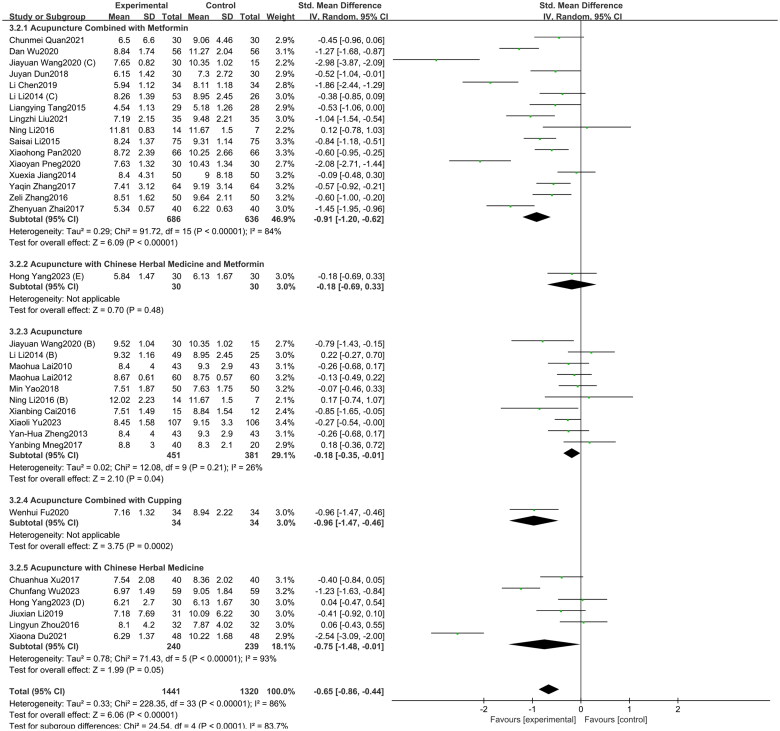
Forest plot depicting the effect of acupuncture and its combined therapies on LH levels in PCOS patients.

The study findings (Figure 3) demonstrate that compared to oral metformin alone, acupuncture and its combined therapies (SMD = −0.65, 95% CI = −0.86 to −0.44, *p* < 0.01, *I^2^* = 86%) can significantly reduce LH levels in PCOS patients. Subgroup analyses were conducted based on intervention types. Due to the limited number of studies for acupuncture with Chinese herbal medicine and metformin, and acupuncture combined with cupping (only one study each), this study only analyzed the subgroup analysis results for acupuncture combined with metformin, acupuncture with Chinese herbal medicine and acupuncture. The results showed that, compared to oral metformin alone, acupuncture combined with metformin (SMD = −0.91, 95% CI = −1.20 to −0.62, *p* < 0.01, *I^2^* = 84%), acupuncture combined with Chinese herbal medicine (SMD = −0.75, 95% CI = −1.48 to −0.01, *p* < 0.01, *I^2^* = 93%), and acupuncture alone (SMD = −0.18, 95% CI = −0.35 to −0.01, *p* < 0.05, *I^2^* = 26%) all contributed to a reduction in LH levels. As depicted in Supplementary Figure 2, the funnel plot indicated a minimal risk of publication bias (Egger’s test *p* = 0.136), and the GRADE analysis determined that the quality of evidence for improving LH was “Moderate” (Supplementary Table 3).

#### 3.2.2. LH/FSH

**Figure 4.**
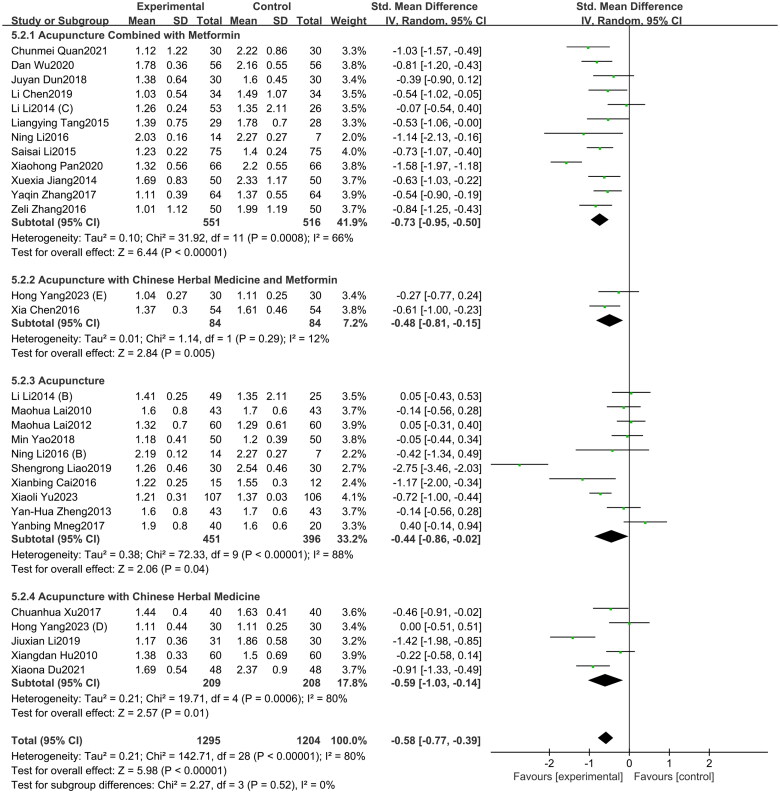
Forest plot depicting the effect of acupuncture and its combined therapies on LH/FSH levels in PCOS patients.

A total of 29 studies included in this study assessed the LH/FSH indicator. Among them, 12 studies involved acupuncture combined with metformin, 10 studies focused on acupuncture alone, five study investigated acupuncture with Chinese herbal medicine, and two studies involved acupuncture with Chinese herbal medicine and metformin. The study findings (Figure 4) demonstrate that compared to oral metformin alone, acupuncture and its combined therapies (SMD = −0.58, 95% CI = −0.77 to −0.39, *p* < 0.01, *I^2^* = 80%) can significantly reduce LH/FSH levels in PCOS patients. Subgroup analyses were conducted based on intervention types. The results indicate that compared to oral metformin, acupuncture combined with metformin (SMD = −0.73, 95% CI = −0.95 to −0.50, *p* < 0.01, *I^2^* = 66%), acupuncture with Chinese herbal medicine (SMD = −0.59, 95% CI = −1.03 to −0.14, *p* = 0.01, *I^2^* = 80%), acupuncture with Chinese herbal medicine and metformin (SMD = −0.48, 95% CI = −0.81 to −0.15, *p* < 0.01, *I^2^* = 12%) and acupuncture (SMD = −0.44, 95% CI = −0.86 to −0.02, *p* < 0.05, *I^2^* = 88%) all showed a greater improvement in LH/FSH levels. As depicted in Supplementary Figure 3, the funnel plot indicated a minimal risk of publication bias (Egger’s test *p* = 0.409), and the GRADE analysis determined that the quality of evidence for improving LH/FSH was “Moderate” (Supplementary Table 3).

#### 3.2.3. T

**Figure 5.**
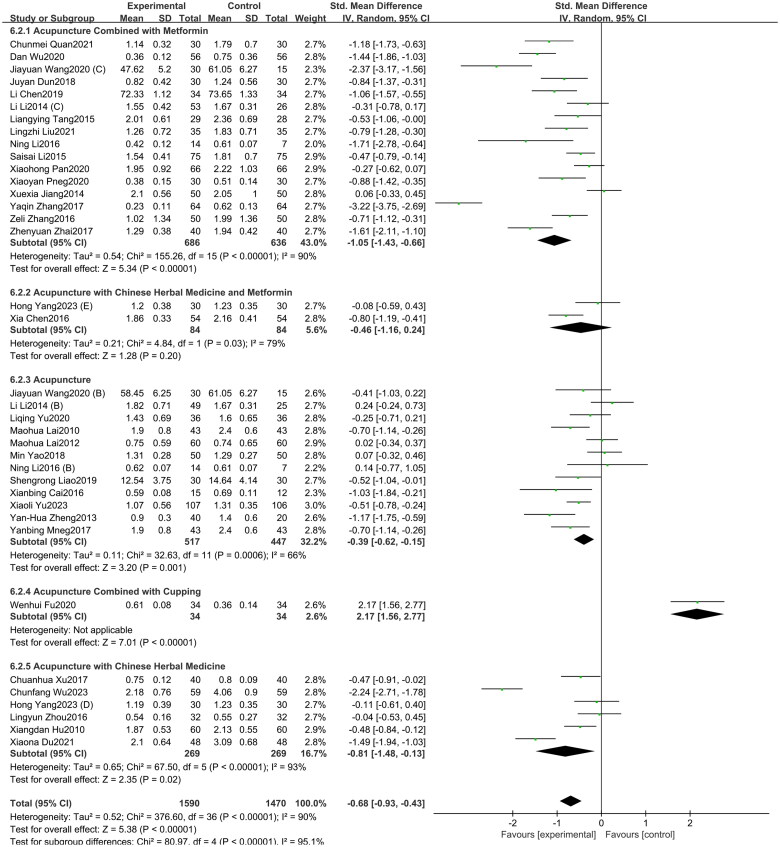
Forest plot depicting the effect of acupuncture and its combined therapies on T levels in PCOS patients.

A total of 37 studies included in this study assessed the T indicator. Among them, 16 studies involved acupuncture combined with metformin, 12 studies focused on acupuncture alone, six study investigated acupuncture with Chinese herbal medicine, two studies involved acupuncture with Chinese herbal medicine and metformin, and one study investigated acupuncture combined with cupping. The study showed (Figure 5) that acupuncture and its combination therapy (SMD = −0.68, 95% CI = −0.93 to −0.43, *p* < 0.01, *I^2^* = 90%) significantly lowered T values in patients with PCOS compared with oral metformin. Since the acupuncture combined with cupping group has only one study, this research only conducted subgroup analyses on the other three intervention groups. Compared to oral metformin alone, acupuncture combined with metformin (SMD = −1.05, 95% CI = −1.43 to −0.66, *p* < 0.01, *I^2^* = 90%), acupuncture combined with Chinese herbal medicine (SMD = −0.81, 95% CI = −1.48 to −0.13, *p* < 0.05, *I^2^* = 93%), and acupuncture alone (SMD = −0.39, 95% CI = −0.62 to −0.15, *p* < 0.01, *I^2^* = 66%) significantly reduced T levels. However, the effect of acupuncture combined with both Chinese herbal medicine and metformin (SMD = −0.46, 95% CI = −1.16 to 0.24, *p* = 0.20, *I^2^* = 79%) was not statistically significant. As depicted in Supplementary Figure 4, the funnel plot indicated a minimal risk of publication bias (Egger’s test *p* = 0.252), and the GRADE analysis determined that the quality of evidence for improving T was “Moderate” (Supplementary Table 3).

### 3.3. Metabolic indicators

#### 3.3.1. FPG

**Figure 6.**
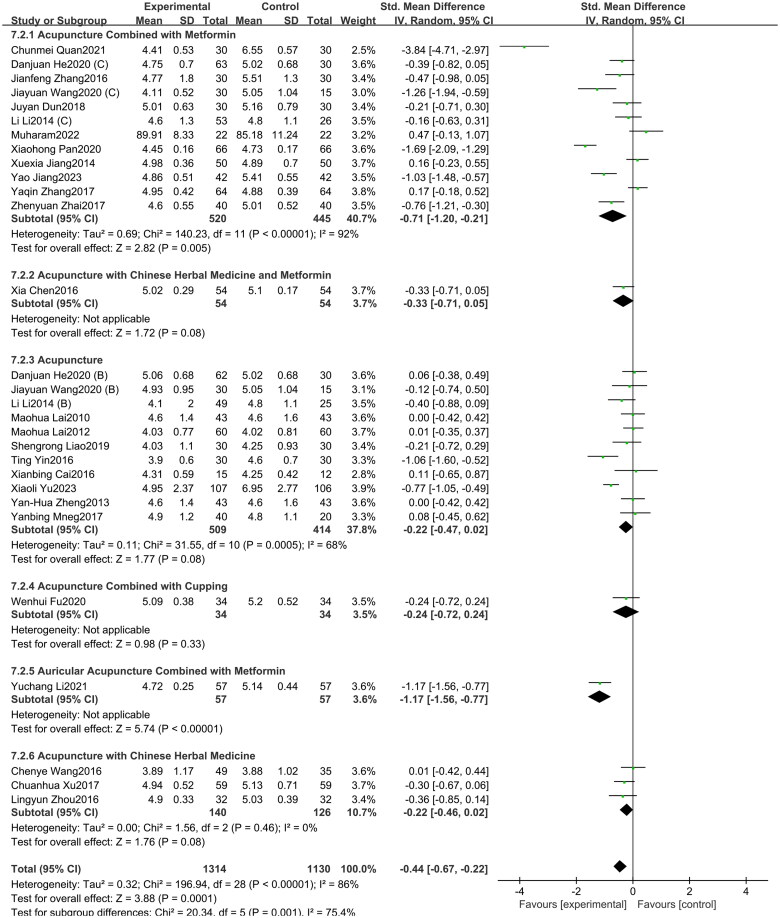
Forest plot depicting the effect of acupuncture and its combined therapies on FPG levels in PCOS patients.

A total of 29 studies included in this study assessed the FPG indicator. Among them, 12 studies involved acupuncture combined with metformin, 11 studies focused on acupuncture alone, three studies involved the acupuncture with Chinese herbal medicine, one study involved acupuncture with Chinese herbal medicine and metformin, one study investigated acupuncture combined with cupping, one study investigated auricular acupuncture combined with metformin. The study showed (Figure 6) that acupuncture and its combination therapy (SMD = −0.44, 95% CI = −0.67 to −0.22, *p* < 0.01, *I^2^* = 86%) significantly lowered FPG values in patients with PCOS compared with oral metformin. Subgroup analysis showed that, compared to oral metformin alone, acupuncture combined with metformin (SMD = −0.71, 95% CI = −1.20 to −0.21, *p* < 0.01, *I^2^* = 92%) significantly reduced FPG levels in patients with PCOS. However, acupuncture combined with Chinese herbal medicine (SMD = −0.22, 95% CI = −0.46 to 0.02, *p* = 0.08, *I^2^* = 0%) and acupuncture alone (SMD = −0.22, 95% CI = −0.47 to 0.02, *p* = 0.08, *I^2^* = 68%) showed no statistically significant difference compared to metformin alone. As depicted in Supplementary Figure 5, the funnel plot indicated a minimal risk of publication bias (Egger’s test *p* = 0.136), and the GRADE analysis determined that the quality of evidence for improving FPG was “Moderate” (Supplementary Table 3).

#### 3.3.2. FINS

**Figure 7.**
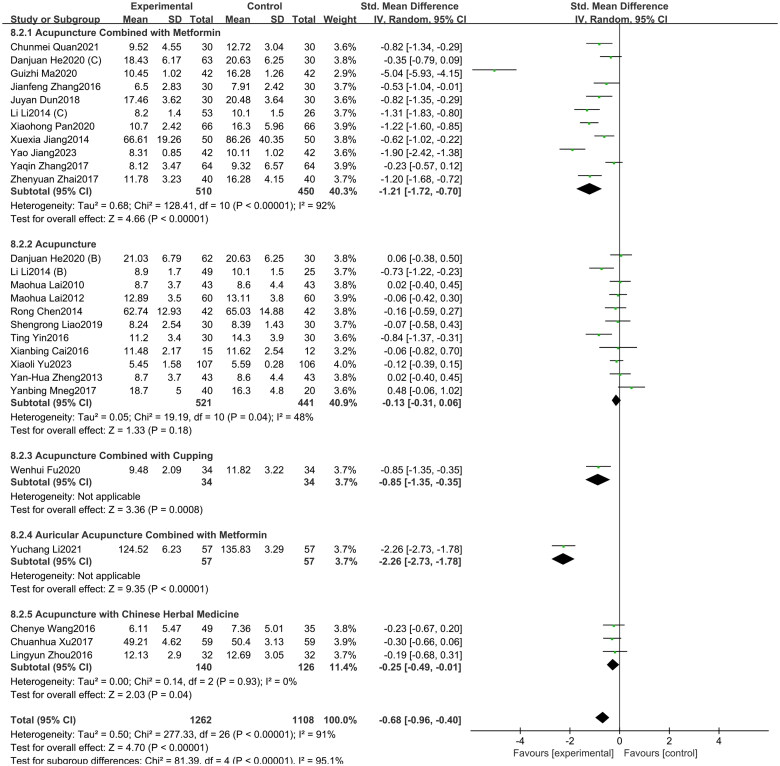
Forest plot depicting the effect of acupuncture and its combined therapies on FINS levels in PCOS patients.

A total of 27 studies included in this study assessed the FINS indicator. Among them, 11 studies involved acupuncture combined with metformin, 11 studies focused on acupuncture alone, three studies involved the acupuncture with Chinese herbal medicine, one study investigated acupuncture combined with cupping, one study involved auricular acupuncture combined with metformin. The study showed (Figure 7) that acupuncture and its combination therapy (SMD = −0.68, 95% CI = −0.96 to −0.40, *p* < 0.01, *I^2^* = 91%) significantly lowered FINS values in patients with PCOS compared with oral metformin. Subgroup analysis showed that, compared to oral metformin alone, acupuncture combined with metformin (SMD = −1.21, 95% CI = −1.72 to −0.70, *p* < 0.01, *I^2^* = 92%) and acupuncture combined with Chinese herbal medicine (SMD = −0.25, 95% CI = −0.49 to −0.01, *p* < 0.05, *I^2^* = 0%) significantly reduced FINS levels. However, acupuncture alone (SMD = −0.13, 95% CI = −0.31 to 0.06, *p* = 0.18, *I^2^* = 48%) showed no statistically significant difference compared to metformin alone. As depicted in Supplementary Figure 6, the funnel plot indicated a significant risk of publication bias (Egger’s test *p* = 0.008), and the GRADE analysis determined that the quality of evidence for improving FINS was “Low” (Supplementary Table 3). A subsequent sensitivity analysis (Supplementary Figure 7) of it revealed that the study by Guizhi Ma (2020) had a substantial impact on the overall effect size. This may be attributed to the exceptionally large SMD reported in this study (SMD = −5.04, 95% CI: −5.93 to −4.15), which significantly deviates from the overall pooled estimate (SMD = −0.53, 95% CI: −0.62 to −0.45).

#### 3.3.3. HOMA-IR

**Figure 8.**
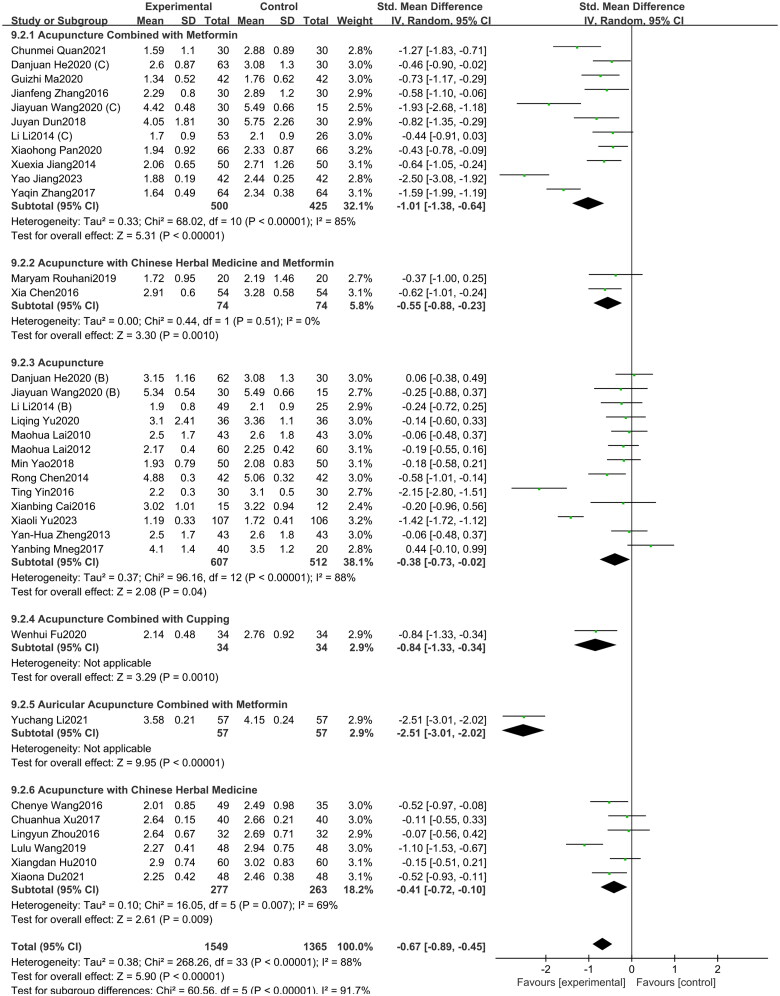
Forest plot depicting the effect of acupuncture and its combined therapies on HOMA-IR levels in PCOS patients.

A total of 34 studies included in this study assessed the HOMA-IR indicator. Among them, 13 studies focused on acupuncture alone, 11 studies involved acupuncture combined with metformin, six studies involved the acupuncture with Chinese herbal medicine, two studies involved acupuncture with Chinese herbal medicine and metformin, one study investigated acupuncture combined with cupping, one study investigated auricular acupuncture combined with metformin. The study showed (Figure 8) that acupuncture and its combination therapy (SMD = −0.67, 95% CI = −0.89 to −0.45, *p* < 0.01, *I^2^* = 88%) significantly lowered HOMA-IR values in patients with PCOS compared with oral metformin. Subgroup analysis shows that, compared to oral metformin alone, acupuncture combined with metformin (SMD = −1.01, 95% CI = −1.38 to −0.64, *p* < 0.01, *I^2^* = 85%), acupuncture combined with both Chinese herbal medicine and metformin (SMD = −0.55, 95% CI = −0.88 to −0.23, *p* < 0.01, *I^2^* = 0%), acupuncture combined with Chinese herbal medicine (SMD = −0.41, 95% CI = −0.72 to −0.10, *p* < 0.01, *I^2^* = 69%), and acupuncture alone (SMD = −0.38, 95% CI = −0.73 to −0.02, *p* < 0.05, *I^2^* = 88%) can significantly reduce HOMA-IR in patients with PCOS. As depicted in Supplementary Figure 8, the funnel plot indicated a minimal risk of publication bias (Egger’s test *p* = 0.268), and the GRADE analysis determined that the quality of evidence for improving HOMA-IR was “Moderate” (Supplementary Table 3).

### 3.4. Weight indicators

#### 3.4.1. BMI

**Figure 9.**
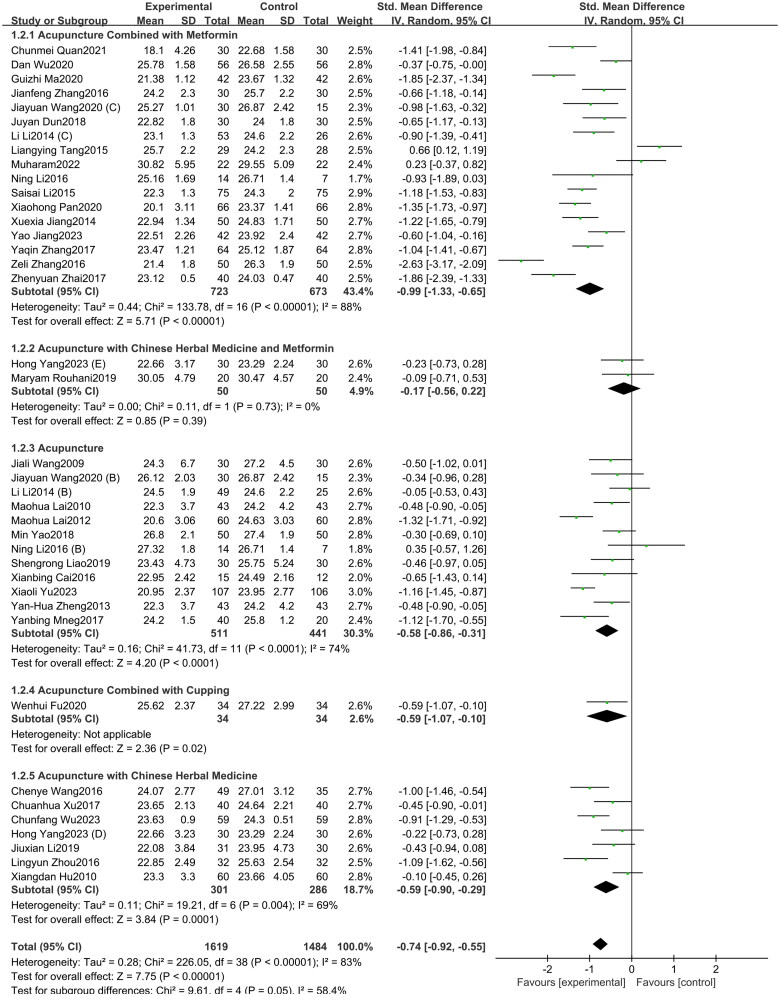
Forest plot depicting the effect of acupuncture and its combined therapies on BMI levels in PCOS patients.

A total of 39 studies included in this study assessed the BMI indicator [72]. Among them, 17 studies involved acupuncture combined with metformin, 12 studies focused on acupuncture alone, seven studies involved the acupuncture with Chinese herbal medicine, two studies involved acupuncture with Chinese herbal medicine and metformin, and one study investigated acupuncture combined with cupping. The study showed (Figure 9) that acupuncture and its combination therapy (SMD = −0.74, 95% CI = −0.92 to −0.55, *p* < 0.01, *I^2^* = 83%) significantly lowered BMI values in patients with PCOS compared with oral metformin. Subgroup analysis showed that Acupuncture with Chinese herbal medicine and metformin (SMD = −0.17, 95% CI = −0.56 to 0.22, *p* = 0.39, *I^2^* = 0%) was found to have no statistical difference compared to taking metformin alone. Additionally, compared to oral metformin alone, acupuncture combined with metformin (SMD = −0.99, 95% CI = −1.33 to −0.65, *p* < 0.01, *I^2^* = 88%), acupuncture with Chinese herbal medicine (SMD = −0.59, 95% CI = −0.90 to −0.29, *p* < 0.01, *I^2^* = 69%) and acupuncture alone (SMD = −0.58, 95% CI = −0.86 to −0.31, *p* < 0.01, *I^2^* = 74%) can significantly reduce the BMI of PCOS patients. As depicted in Supplementary Figure 9, the funnel plot indicated a minimal risk of publication bias (Egger’s test *p* = 0.349), and the GRADE analysis determined that the quality of evidence for improving BMI was “Moderate” (Supplementary Table 3).

#### 3.4.2. WHR

**Figure 10.**
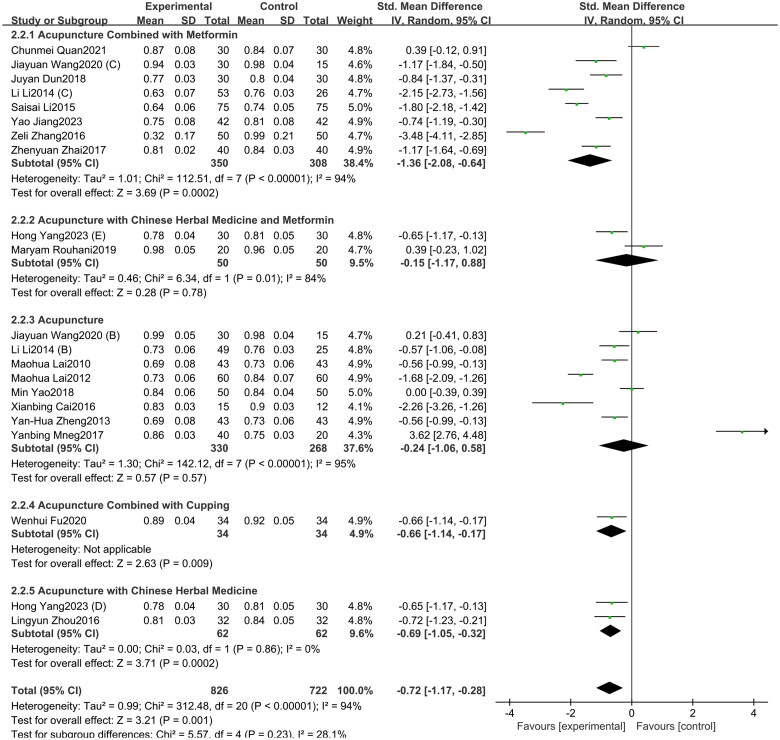
Forest plot depicting the effect of acupuncture and its combined therapies on WHR levels in PCOS patients.

A total of 21 studies included in this study assessed the WHR indicator. Among them, eight studies involved acupuncture combined with metformin, eight studies focused on acupuncture alone, two studies involved acupuncture with Chinese herbal medicine and metformin, two study investigated acupuncture with Chinese herbal medicine, and one study investigated acupuncture combined with cupping. The study showed (Figure 10) that acupuncture and its combination therapy (SMD = −0.72, 95% CI = −1.17 to −0.28, *p* < 0.01, *I^2^* = 94%) significantly lowered WHR values in patients with PCOS compared with oral metformin. Subgroup analysis showed that, compared to oral metformin alone, acupuncture combined with metformin (SMD = −1.36, 95% CI = −2.08 to −0.64, *p* < 0.01, *I^2^* = 94%) and acupuncture combined with Chinese herbal medicine (SMD = −0.69, 95% CI = −1.05 to −0.32, *p* < 0.01, *I^2^* = 0%) significantly reduced WHR levels in patients with PCOS. However, acupuncture alone (SMD = −0.24, 95% CI = −1.06 to 0.58, *p* = 0.57, *I^2^* = 95%) and acupuncture combined with both Chinese herbal medicine and metformin (SMD = −0.15, 95% CI = −1.17 to 0.88, *p* = 0.78, *I^2^* = 84%) showed no statistically significant difference in improving WHR in PCOS patients. As depicted in Supplementary Figure 10, the funnel plot indicated a minimal risk of publication bias (Egger’s test *p* = 0.503), and the GRADE analysis determined that the quality of evidence for improving WHR was “Low” (Supplementary Table 3).

### 3.5. Data mining

**Figure 11.**
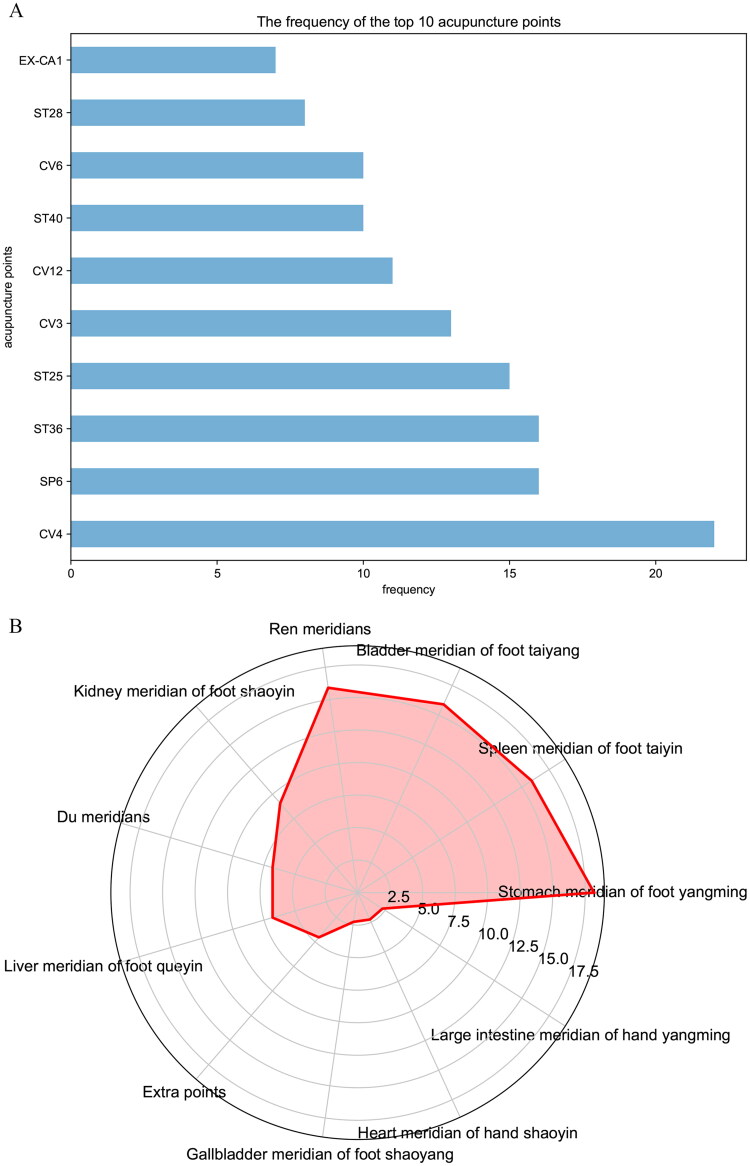
Frequency and meridian analyses of acupuncture points. (A) Frequency analysis of acupuncture points; (B) Meridian attribution analysis.

Due to the overlapping confidence intervals in most subgroup comparisons, it is not possible to determine significant differences between the various interventions. However, based on effect size, acupuncture combined with metformin exhibited the greatest improvement across all indicators. Therefore, this study suggests that acupuncture combined with metformin may offer superior therapeutic benefits for patients with PCOS. To further explore this intervention and provide recommendations for clinical application, a frequency analysis of the acupuncture points used in the acupuncture combined with metformin group was conducted (Figure 11(A)), along with an analysis of their meridian attributions (Figure 11(B)). The study indicates that the Acupuncture Combined with Metformin group involved a total of 44 acupuncture points, among which the CV4 (*n* = 22), SP6 (*n* = 16), and ST36 (*n* = 16) were the most frequently used, suggesting that they may be the most frequently and effectively used acupuncture points for treating PCOS. The analysis of meridian attribution found that the treatment of PCOS mostly utilized points from the Stomach meridian of foot yangming (accounting for 95.45% of all points used), followed by points from the Spleen meridian of foot taiyin and the Bladder meridian of foot taiyang. The results of the association rule analysis (Tables 1 and 2) show that the combination of the CV4 and ST36 is the most common pair of acupuncture points used together (support = 0.71, lift > 1); the combination of CV4, SP6, and ST36 is the most common trio of acupuncture points used together (support = 0.62, lift > 1). Therefore, based on the analysis of these results, this study concludes that the combined use of CV4, SP6, and ST36 is a common and effective choice for acupuncture treatment of PCOS in clinical practice.

**Table 1.** Association rule analysis for pairs of acupuncture points.

Antecedents	Consequents	Support	Confidence	Lift
CV4	ST36	0.71	0.75	1.05
ST36	CV4	0.71	1.00	1.05
SP6	CV4	0.67	1.00	1.05
CV4	SP6	0.67	0.70	1.05
CV3	CV4	0.62	1.00	1.05
CV4	CV3	0.62	0.65	1.05
ST25	CV4	0.62	0.93	0.98
CV4	ST25	0.62	0.65	0.98
SP6	ST36	0.62	0.93	1.30
ST36	SP6	0.62	0.87	1.30

**Table 2.** Association rule analysis for trios of acupuncture points.

Antecedents	Consequents	Support	Confidence	Lift
SP6, CV4	ST36	0.62	0.93	1.30
SP6, ST36	CV4	0.62	1.00	1.05
CV4, ST36	SP6	0.62	0.87	1.30
CV3, CV4	ST36	0.52	0.85	1.18
CV3, ST36	CV4	0.52	1.00	1.05
CV4, ST36	CV3	0.52	0.73	1.18
SP6, CV3	CV4	0.48	1.00	1.05
SP6, CV4	CV3	0.48	0.71	1.15
CV3, CV4	SP6	0.48	0.77	1.15
ST25, CV4	ST36	0.48	0.77	1.08

## 4. Discussion

This study conducted a systematic review and meta-analysis on the effectiveness of acupuncture and its combined therapies in improving symptoms for patients with PCOS. The study included 46 articles, 51 studies, involving 4181 participants. Six types of interventions were examined: acupuncture combined with metformin, acupuncture treatment, acupuncture with Chinese herbal medicine and metformin, acupuncture with Chinese herbal medicine, acupuncture combined with cupping, and auricular acupuncture combined with metformin. The study comprehensively explored the treatment effects of acupuncture and its combined therapies on PCOS by analyzing hormonal indicators (LH, LH/FSH, T), metabolic indicators (FPG, FINS, HOMA-IR), and body weight indicators (BMI, WHR). Hormonal indicators are important measures for diagnosing PCOS, and currently, clinicians often rely on assessing sex hormone levels to diagnose and prognosticate the condition. LH is a hormone secreted by the anterior pituitary gland, playing a crucial role in regulating gonadal function and ovarian steroidogenesis [73]. PCOS patients typically exhibit elevated LH levels; hence, lower LH levels often signify a better treatment response for PCOS. LH and follicle-stimulating hormone (FSH) are gonadotropins secreted by the anterior pituitary gland, and their ratio is considered a sensitive biomarker in the diagnosis of PCOS [74]. In clinical practice, LH levels in PCOS female patients are often reported to be 2-3 times higher than FSH levels [75,76]. T is an androgen that is primarily secreted by the adrenal cortex and ovaries in females [77]. By comparing the concentrations of T, one can assess the androgen excess status in patients with PCOS; the lower the T values, the more evident the treatment effects [78]. Metabolic indicators are commonly used to assess PCOS, and studies have shown that IR is an important etiologic factor in PCOS. Therefore, by testing metabolic indicators (e.g. FPG, FINS, and HOMA-IR), it is possible to assess a patient’s IR status and thus understand the effectiveness of PCOS treatment. FPG refers to the blood glucose level measured after at least 8 h of fasting and is one of the basic biochemical markers used to assess insulin sensitivity [79]. FINS refers to the insulin levels measured in a fasting state [79]. HOMA-IR is a commonly used steady-state model for assessing IR by measuring fasting blood glucose and fasting insulin levels, and it is considered one of the gold standards for evaluating IR [80]. Body Mass Index (BMI) and Waist-Hip Ratio (WHR) are important indicators used to measure an individual’s body shape and obesity status. Most patients with PCOS exhibit overweight or obese conditions [81]. WHR is an indicator to assess the fat distribution of an individual by comparing waist and hip circumference. It has been reported that WHR is positively associated with an increased risk of reproductive disorders in women [82]. The findings indicate that acupuncture and its combined therapies have a significant therapeutic effect on the mentioned indicators for PCOS patients. Subgroup analysis suggests that acupuncture combined with metformin may provide the most significant therapeutic benefits for patients with PCOS. This study further analyzed acupuncture points and their corresponding meridians, revealing that the CV4 is a commonly researched acupuncture point and that the Stomach meridian of foot yangming is often chosen for treating PCOS. Among combinations of two acupuncture points, the pairing of CV4 and ST36 is particularly common; for three-point combinations, CV4, SP6, and ST36 are the most frequently used together. The combination of CV4, SP6, and ST36 has been widely applied in gynecology and reproductive medicine [83–85]. The research indicates that these points are preferred for treating female infertility. Specifically, the CV4 and SP6 are believed to improve uterine microcirculation, while pairing them with ST36 can effectively enhance the endocrine functions of the ovaries and pituitary gland [86].

### 4.1. Therapeutic mechanism

Hyperandrogenism is one of the typical manifestations of PCOS, characterized by elevated levels of free T in the serum. In patients with PCOS, the excessive accumulation of plasma testosterone is converted into estrogens (estrone) in adipose tissue and further transformed into estradiol. This process is often accompanied by abnormal feedback of progesterone, activating the activity of gonadotropin-releasing hormone (GnRH) neurons in the hypothalamus. The primary function of GnRH is to stimulate the anterior pituitary to secrete LH and FSH. When GnRH is excessively activated, it leads to a significant secretion of GnRH peptides and drives an increase in the pulsatile frequency of LH secretion and a relative decrease in the pulsatile frequency of FSH. This series of changes ultimately disrupts the hormonal environment within the ovaries, causing symptoms such as ovulatory dysfunction. Acupuncture is considered to affect the secretion and release of GnRH and gonadotropins in patients with PCOS by modulating the hypothalamic-pituitary-ovarian axis, thereby improving hyperandrogenism. Research has found that acupuncture can stimulate the central sympathetic nervous system, thus regulating the synthesis and release of opioid peptides in the arcuate nucleus of the hypothalamus. This process can affect the levels of β-endorphin in peripheral serum and regulate the production and secretion of GnRH. Additionally, studies have shown that acupuncture can effectively regulate the nerves around the ovaries, restore ovarian morphology, reduce ovarian volume, and improve blood flow [87–90]. Through a meta-analysis of existing experiments, this study has found that acupuncture and its combined therapies can significantly reduce the levels of LH, LH/FSH, and T in patients with PCOS, effectively treating the condition by regulating hormone levels.

In addition to hyperandrogenism, IR has also been found to play a central role in the pathogenesis of PCOS. IR refers to the reduced sensitivity of tissues to insulin. Research has found that excessive insulin in the bodies of PCOS patients can act as an adjuvant gonadotropin, upregulating LH receptor binding sites, thereby stimulating ovarian cells and follicular membrane cells to produce more androgens [73]. Moreover, the accumulation of excessive insulin also affects the pituitary gland and increases GnRH pulse frequency through the MAPK pathway, leading to increased secretion of LH [91]. Acupuncture is considered an effective insulin sensitizer [20,92–94]. This study found that acupuncture and its combined therapies can effectively improve IR symptoms in patients with PCOS, specifically by lowering levels of FPG, FINS, and HOMA-IR. Acupuncture treatment is believed to improve the glucolipid internal environment stability in PCOS patients by regulating glucose tolerance and enhancing insulin sensitivity through the modulation of IRS-1/PI3K/GLUT4 pathway expression [95]. Additionally, research has reported that acupuncture can activate the AMPK pathway to inhibit the expression of SREBP1, thereby regulating symptoms such as hepatic fat accumulation, mitochondrial dysfunction, and IR in PCOS patients [96,97]. Obesity is considered one of the significant reasons for IR in women with PCOS [98,99]. In our study, overweight women (BMI ≥ 25.0) accounted for 98.6% of all patients. Research has found that acupuncture can regulate central nervous system neurons through various pathways to achieve the goals of suppressing appetite and reducing body weight [100]. This study found that acupuncture and its combined therapies can significantly reduce BMI and WHR levels in patients with PCOS. This therapeutic effect might be achieved by reducing mTORC1 activity, which in turn suppresses the expression of neuropeptide Y and agouti-related peptide in the arcuate nucleus [101]. Additionally, acupuncture can decrease the acetylation of FOXO1 through the SIRT1-FOXO1 pathway, thereby stimulating proopiomelanocortin neurons and upregulating the expression of α-MSH [102]. These changes help reduce food intake in PCOS patients, achieving the purpose of weight reduction.

### 4.2. Strength and limitation

This study performed a systematic review and meta-analysis to comprehensively evaluate the effects of acupuncture and its combined therapies on improving hormonal, metabolic, and body weight indicators among PCOS patients. In addition to comprehensively comparing the effectiveness of different treatment options, this study also employed data mining techniques to perform frequency statistics and association rule analysis on the acupuncture points and meridians of the superior intervention group. This revealed the commonly used advantageous acupuncture points in the clinical treatment of PCOS, providing a reference for the specific implementation of acupuncture treatment for PCOS. However, this study also has certain limitations. Firstly, limited by the number of available studies, this research did not differentiate between various types of TCM in the combined treatment with acupuncture but treated TCM as a whole. This approach ignores the potential differences in the effects of different Chinese medicines on disease treatment, which could be a source of heterogeneity. Secondly, some included experiments had small sample sizes and did not clearly report on the ethnicity of participants, which could also contribute to heterogeneity. Lastly, most of the studies included did not report on potential selective reporting, and there was scant reporting on follow-up. Moreover, despite the significant therapeutic effects observed for acupuncture and its combined therapies, this study also identified certain limitations in the included studies. Specifically, a considerable risk of bias was present, particularly due to the uncertainty in blinding procedures. Additionally, our meta-analysis revealed a high degree of heterogeneity in most indicators, which may impact the robustness and generalizability of the findings. Therefore, future experimental studies should focus on addressing these limitations by conducting high-quality, rigorously designed randomized controlled trials to reduce bias and heterogeneity, thereby improving the reliability of evidence and providing stronger support for the clinical application of acupuncture in PCOS treatment.

## Supplementary Material

Supplemental Material

## Data Availability

The data that support the findings of this study are available from the corresponding author, J.C., upon reasonable request.
