# Supplementary material for: Improvement of endocrine and metabolic conditions in patients with polycystic ovary syndrome through acupuncture and its combined therapies: a systematic review and meta-analysis
Source: Ann Med. 2025 Mar 17;57(1):2477295. doi: 10.1080/07853890.2025.2477295 (PMC11915742; doi:10.1080/07853890.2025.2477295)
Supplement: Supplemental Material [file IANN_A_2477295_SM2027.zip › Suppl/Supplementary_Material (19).docx]

Supplementary Material

# 1 Supplementary Table

**Supplementary Table 1** Search strategies.

Search strategy of Embase

| No. | Query |
| --- | --- |
| #4 | #1 AND #2 AND #3 |
| #3 | randomized:ti,ab,kw AND controlled:ti,ab,kw AND trial:ti,ab,kw |
| #2 | acupuncture:ti,ab,kw AND therapy:ti,ab,kw OR (acupuncture:ti,ab,kw AND treatment:ti,ab,kw) OR acupuncture:ti,ab,kw OR (acupuncture:ti,ab,kw AND treatments:ti,ab,kw) OR (treatment,:ti,ab,kw AND acupuncture:ti,ab,kw) OR (therapy,:ti,ab,kw AND acupuncture:ti,ab,kw) OR (pharmacoacupuncture:ti,ab,kw AND treatment:ti,ab,kw) OR (treatment,:ti,ab,kw AND pharmacoacupuncture:ti,ab,kw) OR (pharmacoacupuncture:ti,ab,kw AND therapy:ti,ab,kw) OR (therapy,:ti,ab,kw AND pharmacoacupuncture:ti,ab,kw) OR acupotomy:ti,ab,kw OR acupotomies:ti,ab,kw |
| #1 | polycystic:ti,ab,kw AND ovary:ti,ab,kw AND syndrome:ti,ab,kw OR (ovary:ti,ab,kw AND syndrome,:ti,ab,kw AND polycystic:ti,ab,kw) OR (syndrome,:ti,ab,kw AND polycystic:ti,ab,kw AND ovary:ti,ab,kw) OR ('stein leventhal':ti,ab,kw AND syndrome:ti,ab,kw) OR (stein:ti,ab,kw AND leventhal:ti,ab,kw AND syndrome:ti,ab,kw) OR (syndrome,:ti,ab,kw AND 'stein leventhal':ti,ab,kw) OR (sclerocystic:ti,ab,kw AND ovarian:ti,ab,kw AND degeneration:ti,ab,kw) OR (ovarian:ti,ab,kw AND degeneration,:ti,ab,kw AND sclerocystic:ti,ab,kw) OR (sclerocystic:ti,ab,kw AND ovary:ti,ab,kw AND syndrome:ti,ab,kw) OR (polycystic:ti,ab,kw AND ovarian:ti,ab,kw AND syndrome:ti,ab,kw) OR (ovarian:ti,ab,kw AND syndrome,:ti,ab,kw AND polycystic:ti,ab,kw) OR (polycystic:ti,ab,kw AND ovary:ti,ab,kw AND syndrome:ti,ab,kw AND 1:ti,ab,kw) OR (sclerocystic:ti,ab,kw AND ovaries:ti,ab,kw) OR (ovary,:ti,ab,kw AND sclerocystic:ti,ab,kw) OR (sclerocystic:ti,ab,kw AND ovary:ti,ab,kw) |

This search strategy was adapted accordingly for other electronic databases.

**Supplementary Table 2** Characteristics of the included studies.

| Study, publication  year | No. of patients (treatment/control) | Age  (mean ± SD) | | Intervention | Frequency of acupuncture treatment | Period of treatment | Type of outcome |
| --- | --- | --- | --- | --- | --- | --- | --- |
|  |  | Treatment | Control |  |  |  |  |
| Chenye Wang  2016 | 49/35 | 29.43 ± 4.35 | 29.43 ± 4.35 | Acupuncture with Chinese herbal medicine | 1/2days | 3 months | BMI; FINS; FPG; HOMA-IR |
| Chuanhua Xu  2017 | 40/40 | 24.07 ± 2.26 | 24.42 ± 3.10 | Acupuncture with Chinese herbal medicine | 1/2days | 3 months | LH; LH/FSH; T; HOMA-IR; BMI |
| Chunfang Wu  2023 | 59/59 | 31.70 ± 4.14 | 31.43 ± 4.60 | Acupuncture with Chinese herbal medicine | 1/2days | 3 months | FPG; FINS; LH; FSH; T |
| Chunmei Quan  2021 | 30/30 | 23.80 ± 6.98 | 22.97 ± 6.21 | Acupuncture combined with metformin | 2/week | 3 months | BMI; WHR; FPG; FINS; HOMA-IR; LH; LH/FSH; T |
| Dan Wu  2020 | 56/56 | 28.64 ± 3.73 | 29.43 ± 2.97 | Acupuncture combined with metformin | 2/week | 3 months | BMI; LH; LH/FSH |
| Danjuan He  2020 | 125/60 | (1) 25 ± 7  (2) 24 ± 6 | 25 ± 6 | (1) Acupuncture  (2) Acupuncture combined with metformin | 1/ week | 3 months | FPG; FINS; HOMA-IR |
| Guizhi Ma  2020 | 42/42 | 26 ± 2 | 25 ± 1 | Acupuncture combined with metformin | 1/10days | 3 months | BMI; FINS; HOMA-IR |
| Hong Yang  2023 | 60/60 | (1) 29.13 ± 4.13  (2) 27.87 ± 3.93 | 28.59 ± 3.87 | (1) Acupuncture with Chinese herbal medicine  (2) Acupuncture with Chinese herbal medicine and metformin | 2/ week | 3 months | BMI; WHR; LH; LH/FSH; T |
| Jiali Wang  2009 | 30/30 | 25.8 ± 2.6 | 25.8 ± 2.6 | Acupuncture | 1/2 days | 3 months | BMI |
| Jianfeng Zhang  2016 | 30/30 | 23.36 ± 12.36 | 24.21 ± 13.13 | Acupuncture combined with metformin | 1/15 days | 3 months | BMI; FPG; FINS; HOMA-IR |
| Jiayuan Wang  2020 | 60/30 | 26.43 ± 3.52 | 26.23 ± 3.48 | (1) Acupuncture  (2) Acupuncture combined with metformin | 5/ week | 3 months | LH; T; FPG; HOMA-IR; BMI; WHR |
| Jiuxian Li  2019 | 31/30 | 27.10 ± 3.75 | 28.03 ± 4.32 | Acupuncture with Chinese herbal medicine | 1/15 days | 3 months | BMI; LH; LH/FSH |
| Juyan Dun  2018 | 30/30 | 16.9 ± 1.58 | 17 ± 1.82 | Acupuncture combined with metformin | 2/ day | 3 months | BMI; WHR; LH; T; LH/FSH; FINS; FPG; HOMA-IR |
| Li Chen  2019 | 34/34 | 29.8 ± 3.3 | 30.5 ± 3.5 | Acupuncture combined with metformin | 1/10 days | 3 months | HOMA-IR |
| Li Li  2014 | 102/51 | 27.1 ± 2.5 | 25.2 ± 1.8 | (1) Acupuncture  (2) Acupuncture combined with metformin | 1/ day | 6 months | BMI; WHR; LH; LH/FSH; T; FPG; FINS; HOMA-IR |
| Liangying Tang  2015 | 29/28 | 23.4 ± 12.4 | 24.2 ± 13.1 | Acupuncture combined with metformin | 1/15 days | NA | BMI; LH; LH/FSH; T |
| Lingyun Zhou  2016 | 32/32 | 23 ± 5 | 23 ± 5 | Acupuncture with Chinese herbal medicine | 1/2 days | 3 months | BMI; WHR; FINS; FPG; HOMA-IR |
| Lingzhi Liu  2021 | 35/35 | 27.12 ± 2.04 | 26.95 ± 2.17 | Acupuncture combined with metformin | 1/3 days | 3 months | T; LH |
| Liqing Yu  2020 | 36/36 | 30 ± 6 | 31 ± 6 | Acupuncture | 3/week | 3 months | T; HOMA-IR |
| Lulu Wang  2019 | 48/48 | 32.68 ± 4.82 | 32.75 ± 4.88 | Acupuncture with Chinese herbal medicine | 1/2 days | 3 months | HOMA-IR |
| Maohua Lai  2010 | 43/43 | 26.5 ± 3.0 | 24.9 ± 4.9 | Acupuncture | 2/week | 6 months | BMI; WHR; LH; LH/FSH; T; FPG; FINS; HOMA-IR |
| Maohua Lai  2012 | 60/60 | 26.72 ± 2.65 | 26.46 ± 2.72 | Acupuncture | 1/3 days | 4 months | BMI; WHR; FINS; FPG; HOMA-IR; LH; T |
| Maryam Rouhani  2019 | 20/20 | 27.42 ± 6.09 | 29.82 ± 8.23 | Acupuncture with Chinese herbal medicine and metformin | 2/week | 3 months | BMI; WHR; HOMA-IR |
| Min Yao  2018 | 50/50 | 27.8 ± 4.8 | 28.2 ± 4.5 | Acupuncture | 3/week | 6 months | BMI; WHR; LH; LH/FSH; T; HOMA-IR |
| Muharam  2022 | 22/22 | 27.91 ± 4.09 | 28.14 ± 3.21 | Acupuncture combined with metformin | 3/week | 3 months | BMI; FPG; FINS; HOMA-IR |
| Ning Li  2016 | 28/14 | 28 | 28 | (1) Acupuncture  (2) Acupuncture combined with metformin | 3/week | 6 months | LH; LH/FSH; T; BMI; WHR |
| Rong Chen  2014 | 42/42 | 25 ± 1 | 24 ± 1 | Acupuncture | 1/week | 3 months | FINS; HOMA-IR |
| Saisai Li  2015 | 75/75 | 25.1 ± 2.3 | 24.1 ± 2.2 | Acupuncture combined with metformin | 1/day | 6 months | BMI; WHR; LH; LH/FSH; T |
| Shengrong Liao  2019 | 30/30 | 44.0 ± 6.0 | 43.0 ± 7.0 | Acupuncture | 1/10 days | 3 months | BMI; FPG; FINS; T; LH/FSH |
| Ting Yin  2016 | 30/30 | 27.54 ± 2.03 | 27.56 ± 2.01 | Acupuncture | 1/3 days | 3 months | FPG; FINS; HOMA-IR |
| Wenhui Fu  2020 | 34/34 | 27.23 ± 2.58 | 27.23 ± 2.58 | Acupuncture combined with cupping therapy | 1/day | 3 months | BMI; WHR; LH; T; FPG; FINS; HOMA-IR |
| Xia Chen  2016 | 54/54 | 25.2 ± 4.6 | 25.3 ± 4.3 | Acupuncture with Chinese herbal medicine and metformin | 1/10 days | 3 months | FPG; FINS; HOMA-IR; T; LH/FSH |
| Xianbing Cai  2016 | 15/12 | 25 ± 2.3 | 25 ± 2.3 | Acupuncture | 3/week | 3 months | BMI; WHR; LH; LH/FSH; T; FPG; FINS; HOMA-IR |
| Xiangdan Hu  2010 | 60/60 | 25.27 ± 4.41 | 25.27 ± 4.41 | Acupuncture with Chinese herbal medicine | 1/10 days | 3 months | BMI; T; LH/FSH; HOMA-IR |
| Xiaohong Pan  2020 | 66/66 | 28.6 ± 4.1 | 28.0 ± 4.5 | Acupuncture combined with metformin | 1/10 days | 3 months | BMI; FINS; FPG; HOMA-IR; LH; LH/FSH; T |
| Xiaoli Yu  2023 | 107/106 | 31.13 ± 8.90 | 30.25 ± 9.45 | Acupuncture | 1/2 days | 6 months | BMI; WHR; FPG; FINS; HOMA-IR; LH; T; LH/FSH |
| Xiaona Du  2021 | 48/48 | 29.65 ± 4.32 | 28.20 ± 4.16 | Acupuncture with Chinese herbal medicine | 3/week | 3 months | BMI; HOMA-IR; LH/FSH; T |
| Xiaoyan Peng  2020 | 30/30 | 28.42 ± 1.32 | 28.32 ± 1.35 | Acupuncture combined with metformin | 2-4/week | 3 months | LH; T |
| Xuexia Jiang  2014 | 50/50 | 25.23 ± 1.45 | 26.12 ± 1.02 | Acupuncture combined with metformin | 1/10 days | 3 months | BMI; FPG; FINS; HOMA-IR; LH; LH/FSH; T |
| Yanbing Meng  2017 | 40/20 | 26.5 ± 3.0 | 25.3 ± 4.2 | Acupuncture | 1/3 days | 6 months | BMI; WHR; LH; LH/FSH; T; FPG; FINS; HOMA-IR |
| Yan-Hua Zheng  2013 | 43/43 | 26.5 ± 3.0 | 24.9 ± 4.9 | Acupuncture | 1/day | 6 months | BMI; WHR; LH; LH/FSH; T; FPG; FINS; HOMA-IR |
| Yao Jiang  2023 | 42/42 | 30.68 ± 4.58 | 30.13 ± 4.89 | Acupuncture combined with metformin | 3/week | 3 months | BMI; WHR; FPG; FINS; HOMA-IR |
| Yaqin Zhang  2017 | 64/64 | 28.3 ± 4.2 | 27.9 ± 4.3 | Acupuncture combined with metformin | 1/day | 3 months | FPG; FINS; HOMA-IR; LH; LH/FSH; T |
| Yuchang Li  2021 | 57/57 | 31.42 ± 3.2 | 31.48 ± 3.25 | Auricular acupuncture combined with metformin | 1/5 days | 3 months | FPG; FINS; HOMA-IR |
| Zeli Zhang  2016 | 50/50 | 26.3 ± 4.4 | 27.2 ± 4.1 | Acupuncture combined with metformin | 1/day | 6 months | BMI; WHR; LH; LH/FSH; T |
| Zhenyuan Zhai  2017 | 40/40 | 23.7 ± 2.2 | 23.1 ± 1.9 | Acupuncture combined with metformin | 1/week | 3 months | BMI; WHR; LH; T; FINS; FPG |

**Supplementary Table 3** Assessment according to the GRADE system.

| Quality assessment | | | | | | | Quality | Importance |
| --- | --- | --- | --- | --- | --- | --- | --- | --- |
| No of studies | Design | Risk of bias | Inconsistency | Indirectness | Imprecision | Other considerations |  |  |
| LH | | | | | | | | |
| 34 | Randomised trials | No serious risk of bias | Serious | No serious indirectness | No serious imprecision | None | Moderate | Critical |
| LH/FSH | | | | | | | | |
| 29 | Randomised trials | No serious risk of bias | Serious | No serious indirectness | No serious imprecision | None | Moderate | Critical |
| T | | | | | | | | |
| 37 | Randomised trials | No serious risk of bias | Serious | No serious indirectness | No serious imprecision | None | Moderate | Critical |
| FPG | | | | | | | | |
| 29 | Randomised trials | No serious risk of bias | Serious | No serious indirectness | No serious imprecision | None | Moderate | Important |
| FINS | | | | | | | | |
| 27 | Randomised trials | No serious risk of bias | Very serious | No serious indirectness | No serious imprecision | None | Low | Important |
| HOMA-IR | | | | | | | | |
| 34 | Randomised trials | No serious risk of bias | Serious | No serious indirectness | No serious imprecision | None | Moderate | Critical |
| BMI | | | | | | | | |
| 39 | Randomised trials | No serious risk of bias | Serious | No serious indirectness | No serious imprecision | None | Moderate | Critical |
| WHR | | | | | | | | |
| 21 | Randomised trials | No serious risk of bias | Very serious | No serious indirectness | No serious imprecision | None | Low | Important |

# 2 Supplementary Figures


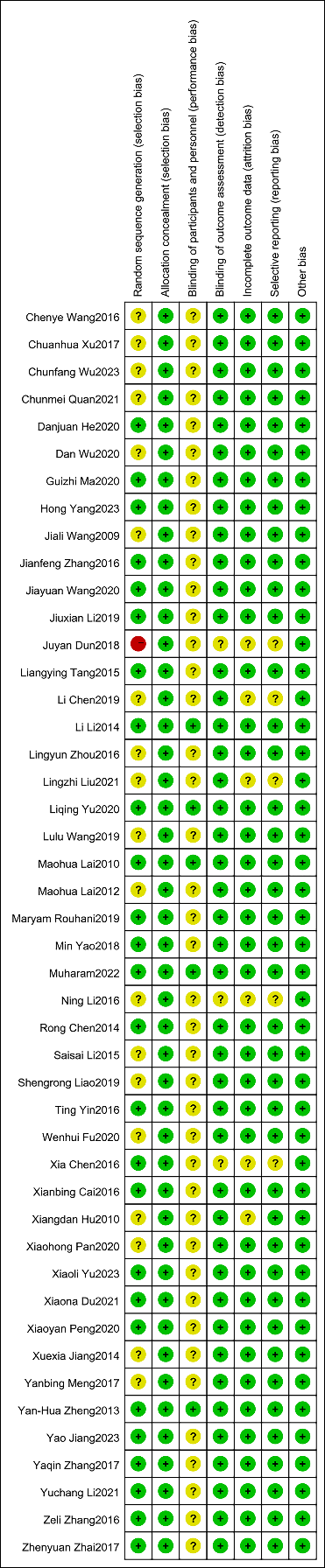


**Supplementary Figure 1.** Risk of bias summary.


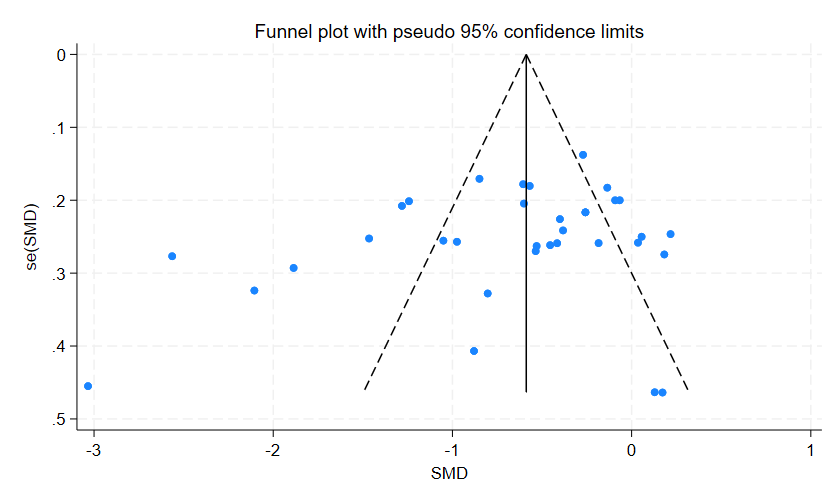


**Supplementary Figure 2.** Funnel plot of acupuncture and its combined therapies on LH levels in PCOS patients.


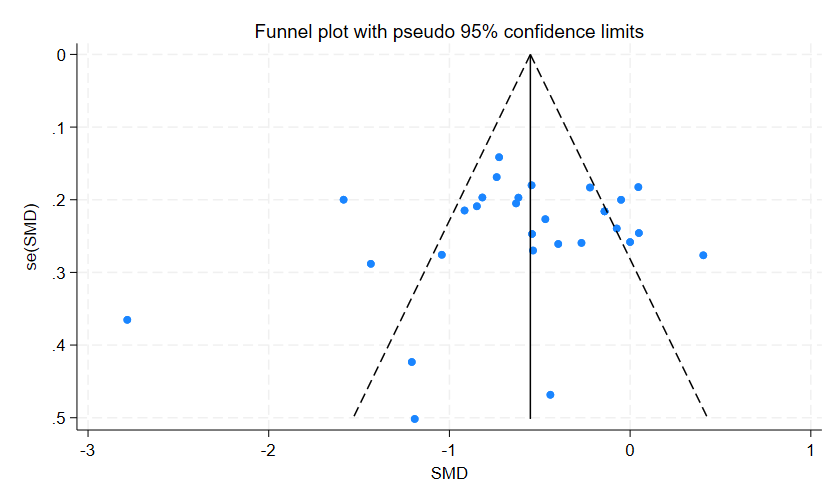


**Supplementary Figure 3.** Funnel plot of acupuncture and its combined therapies on LH/FSH levels in PCOS patients.


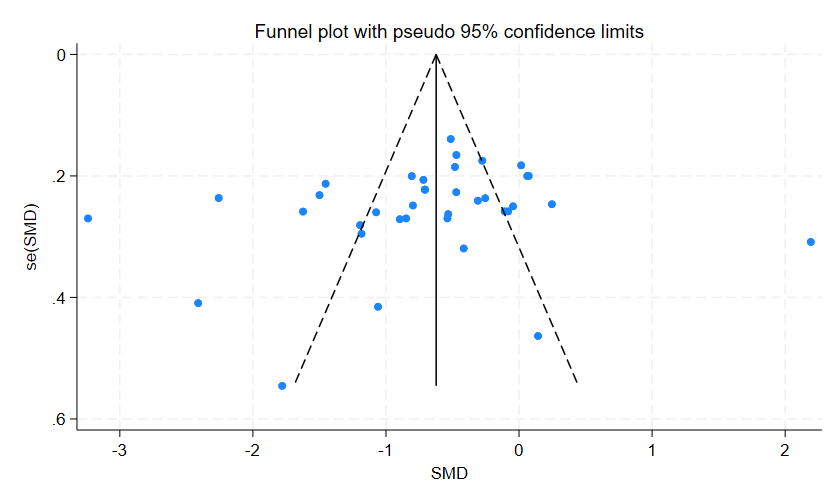


**Supplementary Figure 4.** Funnel plot of acupuncture and its combined therapies on T levels in PCOS patients.


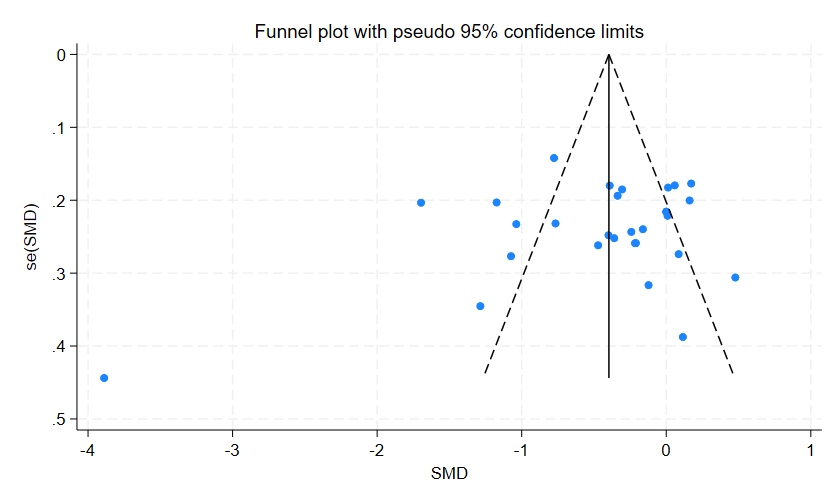


**Supplementary Figure 5.** Funnel plot of acupuncture and its combined therapies on FPG levels in PCOS patients.


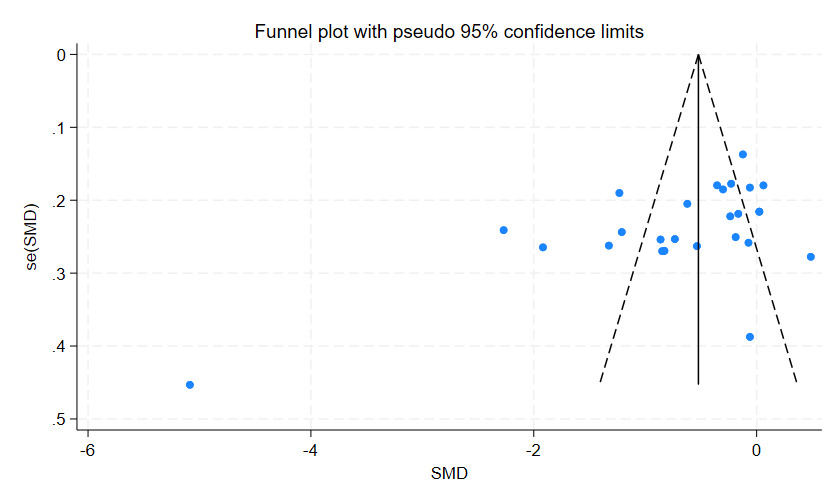


**Supplementary Figure 6.** Funnel plot of acupuncture and its combined therapies on FINS levels in PCOS patients.


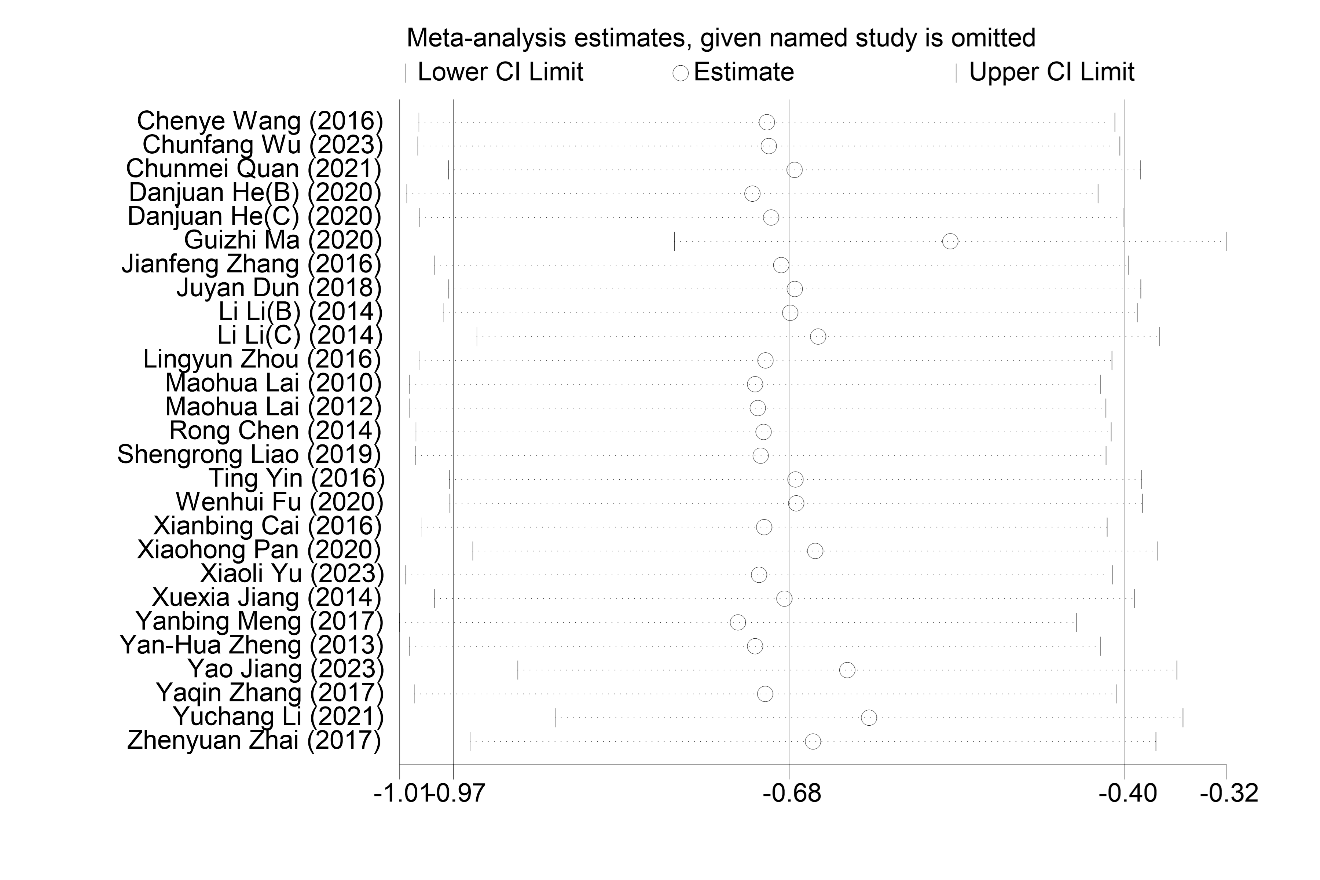


**Supplementary Figure 7.** Sensitivity plot of acupuncture and its combined therapies on FINS levels in PCOS patients.


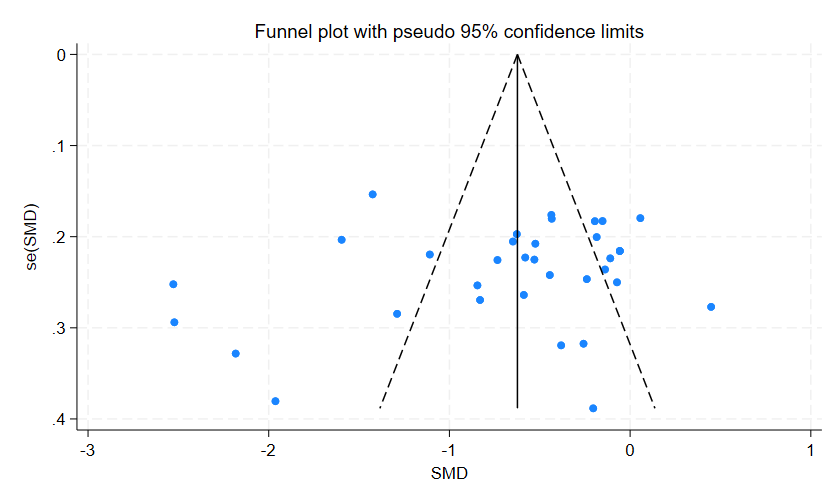


**Supplementary Figure 8.** Funnel plot of acupuncture and its combined therapies on HOMA-IR levels in PCOS patients.


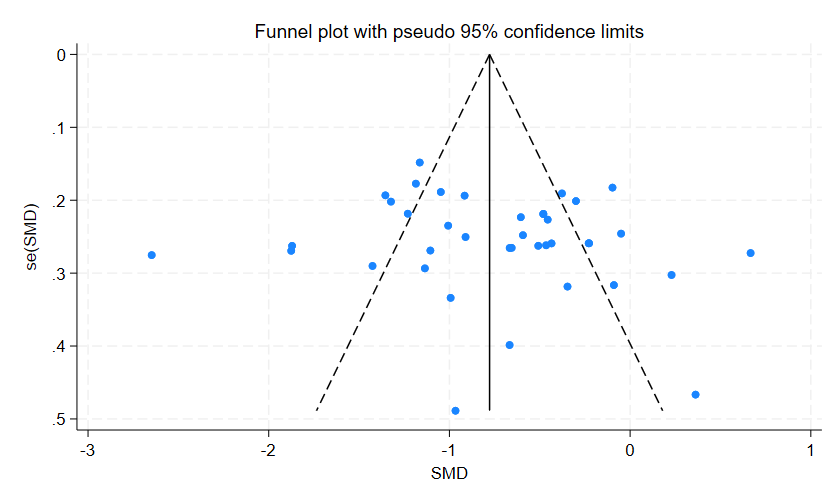


**Supplementary Figure 9.** Funnel plot of acupuncture and its combined therapies on BMI levels in PCOS patients.


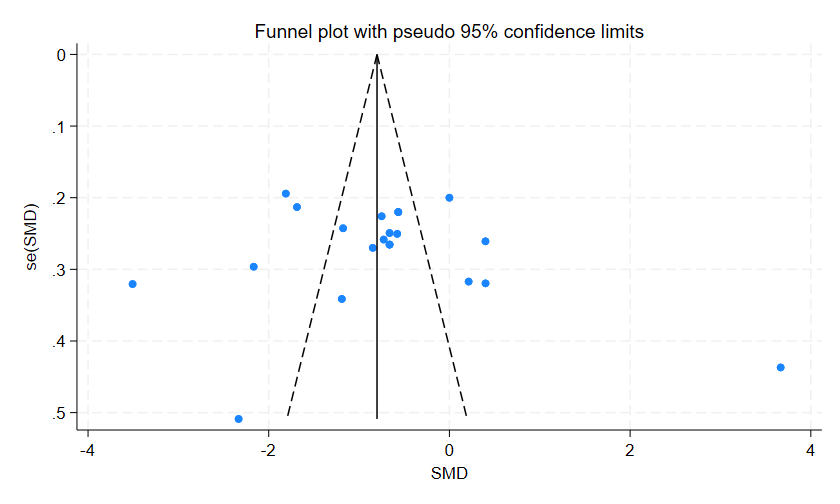


**Supplementary Figure 10.** Funnel plot of acupuncture and its combined therapies on WHR levels in PCOS patients.
